# Cellular Antioxidant Activity of Olive Pomace Extracts: Impact of Gastrointestinal Digestion and Cyclodextrin Encapsulation

**DOI:** 10.3390/molecules25215027

**Published:** 2020-10-29

**Authors:** Kristina Radić, Ivana Vinković Vrček, Ivan Pavičić, Dubravka Vitali Čepo

**Affiliations:** 1 Faculty of Pharmacy and Biochemistry, University of Zagreb, Ante Kovačića 1, 10000 Zagreb, Croatia; kradic@pharma.unizg.hr; 2 Institute for Medical Research and Occupational Health, Ksaverska cesta 2, 10001 Zagreb, Croatia; ivinkovic@imi.hr (I.V.V.); ipavicic@imi.hr (I.P.)

**Keywords:** olive pomace extract, cyclodextrin, in vitro gastrointestinal digestion, antioxidant

## Abstract

Olive pomace is a valuable secondary raw material rich in polyphenols, left behind after the production of olive oil. The present study investigated the protective effect of a polyphenolic extract from olive pomace (OPE) on cell viability and antioxidant defense of cultured human HepG2 cells submitted to oxidative stress induced by *tert*-butylhydroperoxide (tBOOH). The investigation considered possible matrix effects, impact of gastrointestinal digestion and cyclodextrin (CD) encapsulation. Pre-treatment of cells with OPE prevented cell damage and increased intracellular glutathione but did not affect the activity of glutathione peroxidase and superoxide dismutase. OPE matrix significantly enhanced cell protective effects of major antioxidants, such as hydroxytyrosol (HTS), while cyclodextrin encapsulation enhanced activity of OPE against intracellular reactive oxygen species (ROS) accumulation. The obtained results show that OPE is more potent antioxidant in comparison to equivalent dose of main polyphenols (HTS and TS) and that increasing solubility of OPE polyphenols by CD encapsulation or digestion enhances their potential to act as intracellular antioxidants. Antioxidative protection of cells by OPE was primarily achieved through direct radical-scavenging/reducing actions rather than activation of endogenous defense systems in the cell.

## 1. Introduction

Olive pomace is highly accessible, valuable secondary raw material, left behind after the production of olive oil. Its polyphenolic composition is similar to that of olive oil and characterized by a high content of the phenolic alcohol hydroxytyrosol (HTS) and its derivatives tyrosol (TS) and oleuropein (OLE), as the main antioxidative compounds. Their biological activity is strongly correlated to their antioxidant and anti-inflammatory properties since they are able to reduce the pool of reactive oxygen species (ROS) by acting as radical scavengers and metal chelators, as well as to counteract the inflammatory processes associated with the onset and progression of several pathological conditions [1]. The potential of olive polyphenols to protect low density lipoprotein (LDL) from oxidative damage has been well described in scientific literature and is confirmed by the European Food Safety Authority (EFSA) health claim [2]. Although proven to be valuable and low-cost source of HTS and its derivates, olive pomace represents a highly complex matrix that makes an effective extraction procedure a challenging task. Several green and sustainable techniques have been developed and optimized in the last decade resulting in the enhancement of polyphenols’ extraction yields [3,4].

However, raw dry olive pomace extracts (OPEs) are characterized by poor technological properties, such as marked hygroscopicity, intense odor and instability and therefore its usage is still limited. Our previous research suggests that the mentioned shortfalls can be successfully remedied by combining efficient green extraction techniques with the process of cyclodextrin (CD) encapsulation leading to improved extraction yields and achievement of desired OPE characteristics [5,6]. CDs are cyclic compounds, composed of at least six D-(+)-glucopyranoside units that are linked to each other with 1,4-glycosidic bonds. They possess a relatively hydrophobic cavity and a hydrophilic external surface, which enables them to form inclusion complexes with structurally varied compounds. Therefore, they have been increasingly used as an eco-friendly means of recovering polyphenolic substances from complex matrices [7]. Available research suggests that, in addition to modifying extraction yields and physio-chemical properties of dry OPE, application of CD in OPE formulation can have significant impact on functional properties of obtained extracts, particularly on their antioxidant activities. We already demonstrated CDs-mediated enhancement of the OPEs antioxidant activity in different food model systems by increasing significantly their polyphenolic content and probably the stability of antioxidants under oxidative conditions. The tested CDs significantly differed regarding their ability to improve the functionality of OPEs; encapsulation with hydroxypropyl-β-CD (hpbCD) and randomly methylated β-CD provided comparable and the most significant benefits [8]. Similar findings have been reported by other authors who observed that positive effects on antioxidant activity were usually associated with improved solubility of formed inclusion complexes [7,9,10].

Nutraceutical properties of polyphenol rich plant extracts can be additionally modified during gastrointestinal digestion through different mechanisms: chemical degradation, liberation from complex matrix or formation of metabolites. These modifications significantly depend on the polyphenolic profile of the plant extract and the conditions in gastrointestinal tract, which bias detailed prediction of such processes. Previous studies showed that HTS and OLE concentrations remain stable during OPE digestion, while the amount of bioaccessible TS increases significantly in comparison to native, undigested sample [11]. Other authors have also shown that polyphenolic composition of food- or plant-derived extracts and consequently their biological activity is significantly affected by gastrointestinal digestion [12,13,14]. The presence of CDs in the formulation also affected the bioaccessibility of polyphenolic antioxidants by increasing bioaccessibility of TS, probably by formation of inclusion complexes and prevention of TS adhesion to bile salts or other macromolecules. Particular CDs can also directly affect transepithelial permeability of OPE polyphenols, probably through direct effects on Caco-2 cell monolayer [11].

Antioxidant activity of polyphenols is usually attributed to their ability to scavenge free radicals, chelate metals and generally by their redox activity. However, newer experimental data indicate that polyphenols may also offer an indirect protection through activation of endogenous defense systems in the cell. The latest studies strongly suggest that dietary polyphenols can stimulate antioxidant transcription and detoxification defense systems through antioxidant responsive elements inducible by oxidative and chemical stress [15]. To our knowledge, antioxidant activity of OPE polyphenols has not been investigated in cellular model. Even though HTS and TS are believed to be the strongest antioxidants in OPE, there is also no study comparing their activity in the complex OPE matrix to that of pure compounds, in terms of the impact on their activity and the cumulative effect of other antioxidants in OPE.

Therefore, the aim of the present study was to assess the activity of OPE polyphenols against oxidative cell damage induced by *tert*-butyl hydroperoxide (tBOOH) by taking into account the impact of complex OPE matrix and possible polyphenol interactions, encapsulation with hpbCD, and changes in polyphenolic composition induced by gastrointestinal digestion.

## 2. Results and Discussion

In this study, the impact of olive pomace matrix and encapsulation with CD on olive polyphenols’ antioxidative activity was examined in cellular model of oxidative stress. HepG2 cells constitute a validated model to evaluate cellular antioxidative defense system that has been used in many nutritional studies to reveal the mechanisms of polyphenols’ antioxidant activity. Samples used for cell treatment were: native sample (nat) and its bioaccessible fraction (nat_bf); hpbCD sample that contained hydroxypropyl β cyclodextrin and its digest (hpbCD_bf); mix that contained HTS and TS in the same concentration as in native sample; HTS and TS as one-compound samples.

### 2.1. Cell Viability

The cytotoxic effects of OPE and pure polyphenols on HepG2 cells were evaluated by a 3-[4-dimethylthiazole-2-yl]-2,5 diphenyltetrazolium bromide (MTT) assay. The results, shown in Figure S1, reveal no impact on cell viability compared to positive control cells. Therefore, the analyzed extracts and pure compounds were used at the noted concentrations (Table 1) for the examination of the impact on cell’s viability and antioxidative defense system. Similar samples containing olive products’ extracts and pure polyphenols were already reported as non-toxic by several authors in different cell lines [16,17,18].

**Figure 1:**
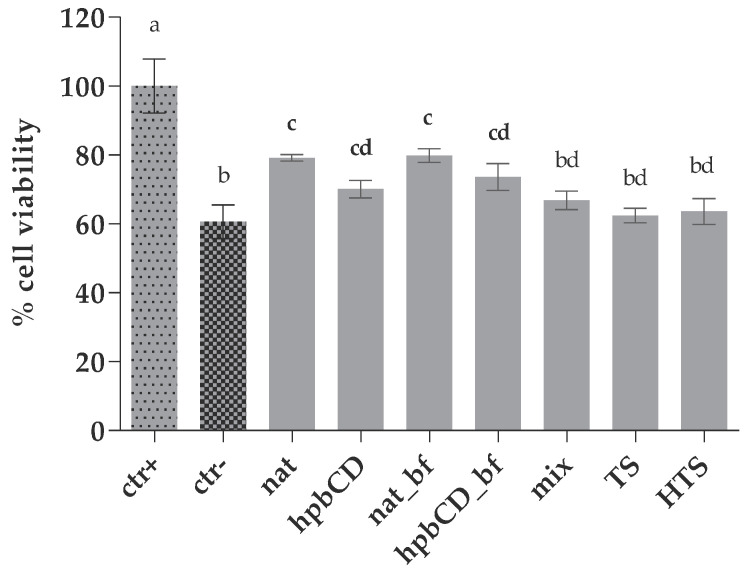
Protective effect of OPEs and pure polyphenols against oxidative stress on HepG2 viability. Data are presented as mean ± standard deviation (*n* = 3) of the percentage calculated according to Equation (1); Positive control cells (ctr+) were treated with PBS while negative control cells (ctr^−^) were treated with tBOOH. All the other cells were treated both with samples and tBOOH. The final concentrations of the samples in wells are noted in Table 1. Different letters (a, b, c, d) indicate statistically significant differences compared to ctr+ (*p* < 0.05). Native olive pomace extract (nat), olive pomace extract with hydroxypropyl β-cyclodextrin (hpbCD); bioaccessible fraction (bf), tyrosol (TS), hydroxytyrosol (HTS), TS and HTS in the same concentration as in native sample (mix).

As shown in Figure 1, the treatment of HepG2 cells with 350 μM tBOOH significantly reduced cell viability clearly showing the toxic effect of peroxide. A significantly lower percentage of viable cells compared to the positive control indicated that pretreatment with analyzed extracts did not eliminate the prooxidative effect of tBOOH completely. This was probably due to the high concentration of tBOOH that was used to fulfil the requirement for clear statistical difference between positive and negative control (0 < z′ < 0.5). However, a significant increase in viability was observed in cells treated with nat, hpb, nat_bf, and hpb_bf samples compared to negative control (Figure 1), while no difference between nat and hpb samples showed that cyclodextrin encapsulation did not affect the antioxidant activity of the analyzed OPEs. On the other hand, mix, TS and HTS samples did not exert protective effects on cell viability at the applied concentrations. Given the fact that mix, HTS and TS were applied in concentrations equivalent to those of nat, observed results indicate that the compounds other than TS and HTS in OPE, such as oleuropein, demethyloleuropein and ligstroside, possess strong antioxidative activity and significantly contribute to antioxidant activity of OPE. To our knowledge, this is the first study comparing antioxidative activity of extracts obtained from olive waste products to antioxidant activity of its main constituents, known as powerful antioxidants, such as HTS and TS. Obtained results also confirm the ability of polyphenols to exert their activity when present in the complex matrix. There was no difference in cell protective effects between digested and undigested OPEs indicating unaltered antioxidative potential during digestion, which is consistent with the results of our previous study where the changes of antioxidant activity of OPE during gastrointestinal digestion were monitored using non-cell based Trolox equivalent antioxidative assay [11]. Also, even though it is known that the additional hydroxyl group at position 3 on the phenol ring provides to HTS stronger antioxidative activity [19], there was no clear difference in TS and HTS effects of on cell viability. That was also probably due to the high concentration of prooxidant.

### 2.2. Reactive Oxygen Species (ROS) Determination

**Figure 2:**
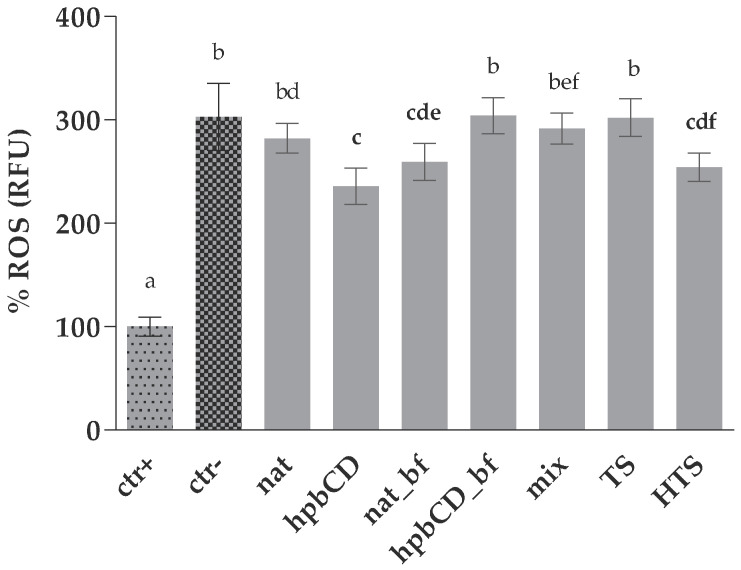
Protective effect of OPEs and pure polyphenols against oxidative stress on HepG2 ROS generation. Data are presented as mean ± standard deviation (*n* = 3) of the relative fluorescence units (RFU) calculated according to Equation (2); Positive control cells (ctr+) were treated with PBS while negative control cells (ctr^−^) were treated with tBOOH. All the other cells were treated both with samples and tBOOH. The final concentrations of the samples in wells are noted in Table 1. Different letters (a, b, c, d, e, f) indicate statistically significant differences compared to ctr+ (*p* < 0.05). Native olive pomace extract (nat), olive pomace extract with hydroxypropyl β-cyclodextrin (hpbCD); bioaccessible fraction (bf), tyrosol (TS), hydroxytyrosol (HTS), TS and HTS in the same concentration as in native sample (mix).

The treatment of HepG2 cells with 50 μM tBOOH significantly increased intracellular ROS, clearly showing the toxic effect of peroxide. ROS are known to play a central role in mediating various diseases-originating processes, such as in cancer and coronary heart disease. Therefore, the elimination of the excessive ROS production and its scavenging is an essential strategy in the prevention of many illnesses. The ability of investigated samples to scavenge ROS was investigated by 2′,7′-dichlorofluorescein diacetate (DCFDA) assay and expressed as relative fluorescence (RFU) (Figure 2). Obtained results showed significantly higher RFU values of samples compared to the positive control indicating that they did not eliminate the prooxidative effect of tBOOH completely. Again, this was also probably due to the high concentration of tBOOH that was used to fulfil the requirement for clear statistical difference between positive and negative control (0 < z′ < 0.5). A significant decrease of RFU compared to negative control was observed at cells treated with hpb, nat_bf and HTS samples (Figure 2). The nat sample was the only OPE that did not decrease intracellular ROS but increased cellular viability (Figure 1). This unexpected result could be explained by methodological differences between the applied methods. In this method, contrary to the cell viability and intracellular GSH level determination (see below), the samples were removed from the wells before adding the prooxidant. Therefore, the measured antioxidative effect can be attributed only to the compounds that entered the cell during incubation period. It could be concluded that the ability of antioxidants to cross the cell membrane and to exert their activity intracellularly was the lowest in undigested native OPE. This is in consistence with our previous results that showed positive impact of gastrointestinal digestion on bioaccessibility of OPE polyphenols, and positive impact of cyclodextrin encapsulation of transcellular passage of OPE antioxidants [11]. Observed effects can be explained by the fact that OPE polyphenols, including HTS, are often found as part of larger complex molecules, such as oleuropein or hydroxytyrosol 4-β-D-glucoside which also have strong antioxidative properties [20]. However, due to their sizes and polarities, the cellular uptake of these compounds is usually limited by glucose transporters and is lower than the uptake of their aglycone [21]. The mix also did not show protective effect on ROS production confirming that TS and HTS are not able to annul an intracellular deleterious effect of tBOOH in concentrations that are found in OPE. Interestingly, the hpb-containing sample showed antioxidative effect contrary to its bioaccessible fraction. Since hpb was already reported as small molecule extraction enhancer from OPE matrix [8,22], it probably increased the yield of small bioactive molecules during extraction. It seems that those smaller compounds were more susceptible to degradation during the digestion process as the antioxidative potential decreased in hpb_bf. According to Wojtunik-Kulesza [23], small polyphenols are more prone to pro-oxidation during digestion than large-molecular-weight compounds. Processes that can lead to pro-oxidation of polyphenols include the presence of metal ions, oxygen molecules, as most of all alkali pH. The latter was already reported as the main culprit for decreased bioaccessibility of small polyphenols including caffeic and rosmarinic acid [24]. Digestion of OPE resulted in unaltered concentrations of HTS and an increase of TS concentration (Figure S2) indicating that observed degradation may be linked to other small antioxidants from OPE such as ferulic and vanillic acid [25], which should be confirmed by future, targeted investigation.

The TS on the other hand was reported as an effective cellular antioxidant, probably due to its intracellular accumulation [19]. However, we were unable to confirm that postulate not even with the increased concentration of TS, observed during digestion. Unlike in the case of the cell viability, HTS sample (in a concentration approximately five times higher than in mix sample) decreased the ROS production while TS did not. It seems that the presence of HTS in concentration of 20 µM could eliminate the intracellular effect of 50 µM tBOOH as was obvious from the ROS measurement. Treatment with 350 µM tBOOH limited the utility of cell viability assay to observe the protective effects of polyphenols in lower concentrations. These results confirm the importance of using multi-method approach to examine the antioxidative properties [26,27]. The TS in concentration of 20 µM (approximately 17 times higher concentration than in OPE) failed to protect the cells from prooxidative effect of 50 µM tBOOH in this method. In the already mentioned work from Di Benedetto [19], TS showed intracellular accumulation and scavenging of ROS, but at a concentration of 500 µM, i.e., 25 times higher than the one we used in our assay. Therefore, the antioxidative effect of TS seems to be a dose- and incubation time-dependent.

### 2.3. GSH Determination

**Figure 3:**
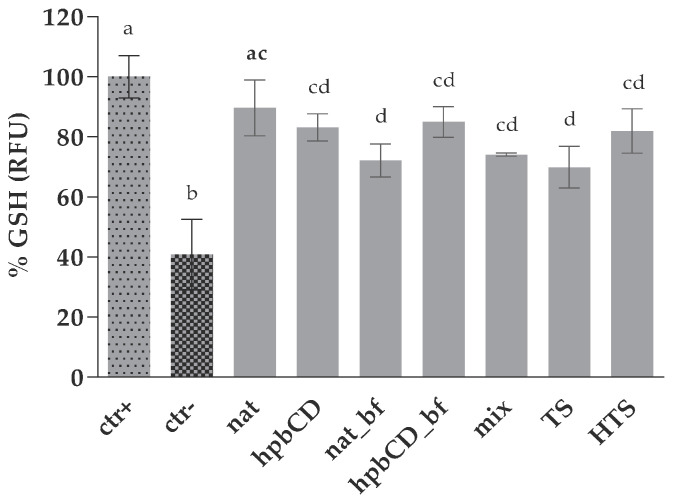
Protective effect of OPEs and pure polyphenols against oxidative stress on HepG2 GSH content. Data are presented as mean ± standard deviation (*n* = 3) of the relative fluorescence units (RFU) calculated according to Equation (2); Positive control cells (ctr+) were treated with PBS while negative control cells (ctr^−^) were treated with tBOOH. All the other cells were treated both with samples and tBOOH. The final concentrations of the samples in wells are noted in Table 1. Different letters (a, b, c, d) indicate statistically significant differences compared to ctr+ (*p* < 0.05). Native olive pomace extract (nat), olive pomace extract with hydroxypropyl β-cyclodextrin (hpbCD); bioaccessible fraction (bf), tyrosol (TS), hydroxytyrosol (HTS), TS and HTS in the same concentration as in native sample (mix).

GSH is the main intracellular antioxidant and works as the substrate in GP-catalyzed neutralization of organic peroxides. Our results obtained be performing monochlorobimane (mBCl) assay showed that the treatment of HepG2 cells with 50 μM tBOOH significantly decreased intracellular GSH level clearly showing the toxic effect of peroxide while all tested samples succeeded to annul this effect as visible in an increase of RFU compared to the negative control (Figure 3). Interestingly, both nat and mix sample showed protective effect on GSH preservation, contrary to the increase in ROS level as observed by DCFDA assay. The difference could be explained by the fact that the samples were co-incubated with prooxidant in this method; so, both intra- and extra-cellular antioxidative activities against 50 µM tBOOH were measured. That observation confirms an ability of TS and HTS to scavenge the prooxidant in concentrations found in OPE and to maintain GSH in reduced form, which is in line with several previous in vitro- and in vivo studies. A study on healthy volunteers by Visoli [28] showed that polyphenols from olive mill waste waters increased total plasma glutathione. Pereira-Caro [29] on the other hand proved the ability of HTS in concentration 0.5 µM (approximately seven times lower concentration than the one found in OPE) to annul the deleterious effect of tBOOH at concentration of 400 µM, what is an eight times higher concentration than the concentration used in our assay. Therefore, the presence of HTS in OPE along with other antioxidants seem to be enough to exert their antioxidative activity against high concentrations of prooxidants. Cyclodextrin encapsulation did not affect the extent of their potential in decreasing oxidative stress as there was no difference between nat and hpb samples. The protective effect of HTS on GSH content was stronger than the effect of TS, as well as in ROS determination assay. However, the difference in GSH assay was not statistically significant.

### 2.4. GPx and SOD Activity Determination

GPx and SOD play the crucial role in the defense against oxidative stress. SOD destroys highly reactive superoxide to hydrogen peroxide and oxygen. Hydrogen peroxide is further neutralized by GPx into oxygen and water by utilizing GSH as substrate.

**Figure 4:**
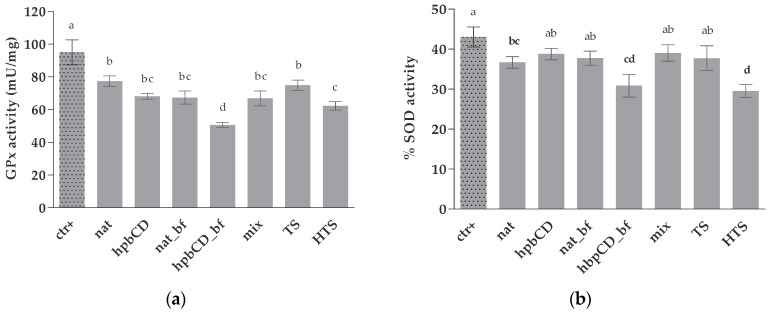
Effect of OPEs and pure polyphenols on enzyme activity. Data are presented as mean ± standard deviation (*n* = 3) of the (**a**) GPx activity calculated according to Equation (3) and (**b**) SOD activity calculated according to Equation (4); Positive control cells (ctr+) were treated with PBS. The final concentrations of the samples in wells are noted in Table 1. Different letters (a, b, c, d) indicate statistically significant differences compared to ctr+ (*p* < 0.05). Native olive pomace extract (nat), olive pomace extract with hydroxypropyl β-cyclodextrin (hpbCD); bioaccessible fraction (bf), tyrosol (TS), hydroxytyrosol (HTS), TS and HTS in the same concentration as in native sample (mix).

In this study, cells pretreated with OPEs showed significantly lower GPx activity in comparison to positive control (treated only with PBS). All analyzed samples caused similar decrease in enzyme activity, with exception of bioaccessible fraction of hpb, which showed the strongest inhibition (Figure 4a). SOD activity was generally unaffected by tested OPEs and it was significantly reduced only in cells pretreated nat, hpb_bf, and HTS. Again, hpb_bf had the highest impact in lowering enzyme’s activity (Figure 4b).

Activity of SOD and GPx changes depending on the redox state of the cell. In vivo, their activity generally increases in response to oxidative stress, but if the stress continues, it is possible that the cell mechanisms will stop coping with persisting stress state, and in those late stages, the activity of enzymes will start do decrease. For example, extremely high concentrations of superoxide radicals in the cell will cause depletion of SOD and it will become unable to fight against free radicals. Therefore, polyphenols can affect the activity of mentioned enzymes indirectly, by affecting the redox state of the cell.

However, dietary polyphenols can also exert direct effects on the expression of genes regulating transcription of enzymes involved in antioxidant defense of the cell, acting similarly to numerous endogenous transcriptional factors [30]. For example, t-butylhydroquinone, has been identified as xenobiotic that can activate particular promotor regions called antioxidant responsive elements (ARE) and induce transcription of particular detoxifying, metal-binding or antioxidative enzymes. ARE activating effects have been proven for different fruit and vegetable extracts rich in polyphenols and carotenoids, and in those studies their ARE activating ability didn’t correlate with direct radical scavenging or reducing potential [31,32].

The impact of OPE on the activity of antioxidative enzymes has not been investigated so far. However, Marinić and co-authors [33] showed that preexposure to olive oil polyphenols affected endogenous cellular defense mechanisms via the stress response gene-profiles associated with hepatoprotection in the model of liver regeneration induced by one-third hepatectomy. Leskovec and co-workers [34] investigated the effect of olive leaf extract on the activity of GPx and SOD in animal model and found no changes in blood SOD activity while observed effects on GPx activity varied depending on the applied diet matrix. The results of the mentioned studies indicate different complex mechanism of involvement of olive polyphenols into regulation of oxidative response in vivo and emphasize the importance of the length of exposure to extracts, dosage and the existence of specie- and tissue- specific effects probably as the consequence of differences in bioavailability and metabolism pathways. Therefore, it is impossible to discuss data obtained in vitro in relation to available results of animal studies. The inhibitory effect of OPEs on the activity of GPx and SOD in cell lines tested here could be explained by their direct radical scavenging activities causing the decrease of intracellular ROS.

In conclusion, this study showed that a pretreatment of cells with OPE increased intracellular GSH and prevented radical-induced cell damage. In comparison to HTS, TS or their mix, OPE showed significantly higher ability to protect viability of cell subjected to treatment with free radicals, indicating significant contribution of other compounds in OPE (oleuropein, demethyloleuropein and ligstroside) to its antioxidative potential. The hpb, nat_bf and HTS showed the highest intracellular radical scavenging activity; however, measured antioxidative effect of polyphenols was dose- and incubation time-dependent and can vary significantly depending on the applied experimental conditions. Cells pretreated with OPEs showed significantly lower GPx activity, while SOD activity was generally unaffected indicating that OPE polyphenols do not exert direct effects on tested enzymes, but can change the redox state of the cell through multiple mechanisms (reductive potential, radical scavenging), resulting in consequential change of SOD/GPx activity.

## 3. Materials and Methods

### 3.1. Samples

Olive pomace (OP) was collected from several two-phase mills in Croatia during autumn 2018. OP was kept at −20 °C (Beko CN161220X, Istanbul, Turkey) in polypropylene bags until use. Pre-treatment included drying at 60 °C for 24 h in an incubator (INKO, Zagreb, Croatia), shredding and sieving on φ 0.8 mm sieve (Prüfsieb DIN 4188, Kassel, Germany), defatting (~5 g of the sample was defatting for 2 h with petrol ether) using the Soxhlet apparatus (INKO SK6ESS, Zagreb, Croatia). Polyphenols were extracted with 20% ethanol from defatted OP (20 g/L) without (native sample) or with the addition of 8 g/L of hydroxypropyl β-cyclodextrin (hpbCD) according to previously optimized procedure [6]. The extraction was performed on 700 W of microwave power in high performance microwave digestion unit (Milestone 1200 mega, Sorisole, Italy) for 10 min. The extracts were cooled on ice and filtered to remove the crude parts. hpbCD was chosen according to the number of successful applications for olive polyphenols encapsulation in literature data. Obtained extracts were freeze dried for 48 h in a lyophilizer (Alpha 1–4 LOC-1, Martin Christ Gefriertrocknungsanlagen GmbH, Osterode am Harz, Germany). In addition, polyphenols specific for OP (hydroxytyrosol (HTS) and tyrosol (TS)) were included in this study mixed (in concentrations that are found in OPE) and as one-compound samples (220 µM). Stock solutions of pure compounds were prepared in DMSO (2 mg/mL) and diluted with PBS.

### 3.2. Reagents

Petrol ether was purchased from Carlo Erba Reagents (Barcelona, Spain) while ethanol and dimethyl sulfoxide (DMSO) were from Gram Mol (Zagreb, Croatia). Hydroxypropyl β-cyclodextrin was purchased from Wacker–Chemie GmbH (Burghausen, Germany). Methanol (≥99.9%) and sodium acetate used for chromatographic analysis were from Sigma–Aldrich (St. Louis, MO, USA) while acetonitrile (≥99.9%) was from Honeywell (Charlotte, NC, USA), and acetic acid from Kemika (Zagreb, Croatia). Reference standards of phenolic compounds 3-hydroxytyrosol (HTS) and tyrosol (TS) were of analytical grade (≥98%) and purchased from Sigma–Aldrich (St. Louis, MO, USA) as well as *tert*-butyl hydroperoxide (tBOOH). Dulbecco’s Phosphate Buffered Saline (PBS liquid, sterile filtered, without calcium, without magnesium, suitable for cell culture) was from Lonza (Basel, Switzerland). Bile salts, pancreatin from porcine pancreas (4 × USP), and Minimum Essential Eagle’s Medium (MEM with Earle′s salts, L-glutamine and sodium bicarbonate, sterile filtered, suitable for cell culture) were purchased from Sigma–Aldrich (St. Louis, MO, USA). Rabbit gastric extract (RGE > 25 U/mg) was from Lipolytech (Marseille, France). Heat inactivated fetal bovine serum (FBS), nonessential amino acids (NEAA), and trypsin were purchased from Capricorn Scientific (Ebsdorfergrund, Germany). 3-[4,5-Dimethyl-thiazole-2-yl]-2,5 diphenyltetrazolium bromide (MTT) was from Panreac AppliChem (Darmstadt, Germany). Fluorescent dyes 2′,7′–dichlorofluorescin diacetate (DCFDA) and monochlorobimane (mBCl) were from Sigma–Aldrich (St. Louis, MO, USA). Glutathione Peroxidase Assay Kit (GPx) was from Cayman Chemical (Ann Arbor, MI, USA) while superoxide dismutase (SOD) assay kit, bicinchoninic acid (BCA), and bovine serum albumin (BSA) were from Sigma–Aldrich (St. Louis, MO, USA). Ultrapure water (18 MΩ) was obtained from SG Reinstwassersystem Ultra Clear UV Plus coupled with SG Wasservollentsalzer-Patrone SG-2800 (Günzburg, Germany). Acqua pro injectione was obtained from the Croatian Institute of Transfusion Medicine (Zagreb, Croatia).

Cells were lysed with a lysis buffer that was made as follows: 25 mL of Solution 1, 250 µL of Solution 2 and 1 tablet of the Complete Protease Inhibitor (Roche Diagnostics, Risch-Rotkreuz, Switzerland). Solution 1 was composed of 50 mM Tris, pH 8.0 (VWR International, Radnor, PA, USA), 137 mM NaCl (Kemig, Zagreb, Croatia), 1% Triton X-100 (Sigma–Aldrich, St. Louis, MO, USA), and 10% glycerol (Gram Mol, Zagreb, Croatia). Solution 2 was composed of 100 mM sodium orthovanadate (Sigma–Aldrich, St. Louis, MO, USA) and hydrogen peroxide (Gram Mol) in a final concentration of 3.6%.

All the solvents needed for chromatographic separation were degassed before analysis with Branson 1210 Ultrasonic Cleaner (Danbury, CT, USA). Acetate buffer was prepared by mixing sodium acetate 0.1 M: acetic acid 0.1 M (2:1 *v*/*v*) and adjusting the pH to 5 with pH meter (702 SM Titrino, Metrohm, Herisau, Switzerland).

### 3.3. Quantification of Phenolic Components by HPLC-FLD

HTS and TS were identified and quantified by an Alliance 2695 HPLC system (Waters, Milford, MA, USA) coupled with a 2475 Multi λ detector (FLD) with Xenon lamp, according to modified method of Tsarbopoulos and co-workers [35]. Samples were prepared by filtration through 0.45 µm PES syringe filters (Macherey–Nagel, Düren, Germany). Chromatographic separation was conducted by injecting 20 µL of sample on a reversed phase column (250 × 4.6 mm, 5 µm) (Agilent Zorbax Eclipse Plus C18, Santa Clara, CA, USA). Mobile phases were 0.05 M sodium acetate buffer pH 5 and acetonitrile with the flow rate of 1 mL/min. Elution was conducted over 20 min at 25 °C. Identification was performed with FLD set at the excitation wavelength of 280 nm, and emission wavelength of 316 nm. Polyphenols were identified by comparing the retention times of the eluting peaks with those of the standards. Peaks were quantified by using the Empower2 software (Waters, Milford, MA, USA) and compared to external standard calibration. Standard stock solutions were prepared by dissolving reference compounds in DMSO. Aliquots of these solutions were further diluted with ultrapure water to obtain calibration curve (1–81 mg/L).

### 3.4. In Vitro Simulated Gastrointestinal Digestion

Bioaccessible fractions (bf) of the OP polyphenols were obtained by in vitro static simulation of gastrointestinal digestion in the upper tract following the standardized protocol described by Brodkorb and co-workers [36]. The procedure was consisted of two sequential incubations; initially in simulated gastric fluid (SGF)/pepsin/gastric lipase to simulated gastric conditions followed by simulated intestinal fluid (SIF)/bile salts/pancreatin to simulate duodenal digestion. Briefly, 500 mg of OPE were dissolved in 2 mL of ultrapure water and mixed with SGF. The samples were incubated at 37 °C for 2 h in a water bath (Büchi B-490, Flawil, Switzerland) with uniform shake at 110 rpm. Then SIF was added and incubated under the same conditions for 2 h. Total volume of the reaction mixture was approximately 18 mL, so the final concentration of extracts was 28 mg/mL. Enzyme solutions were prepared just before use. Samples were put on ice for 10 min and centrifuged (Heraeus Biofuge Stratos, Hanau, Germany) for 20 min at 4 °C and 4100 rpm in order to remove the crude parts of the sample. Supernatants were collected and the enzyme inactivation was done by a heat shock for 5 min at 100 °C in Thermomixer R (Eppendorf, Hamburg, Germany). Then, the samples were cooled in an ice bath for 10 min and centrifuged again under the same conditions. Supernatants, that represent bioaccessible fraction (bf) of the samples, were collected and stored at −80 °C until analyses.

### 3.5. Cell Culture

For investigation of antioxidative effect human liver cancer cell line (HepG2) was used. HepG2 cells (American Type Culture Collection (ATCC), Manassas, VA, USA) were cultured in MEM supplemented with 10% heat-inactivated FBS and 1% A/A. Cell cultures were maintained at 37 °C, in a humidity saturated atmosphere consisted of 5% CO_2_ (Sanyo MCO-20AIC CO_2_ Incubator, Osaka, Japan). Medium was changed every 2–3 days. Cells were passaged at 80–90% confluence.

Samples used for cell treatment were prepared as follows: native sample (nat) and its bioaccessible fraction (nat_bf) contained 9 mg/mL of OPE without added CD; hpbCD sample and its digest (hpbCD_bf) contained OPE with CD in concentration of 28 mg/mL; mix contained HTS and TS in the same concentration as in native sample, i.e., 41 µM and 13 µM respectively (data obtained with HPLC analysis (nat OPE chromatograph is shown in Figure S3)); HTS and TS were one-compound samples and contained 220 µM of the analyte. A concentration of OPE in samples was chosen by taking into the account the total phenolic content obtained by analyzing samples with standard Folin-Ciocalteu method in order to annul the dilution of the OP by CD (Figure S4). The analysis revealed that the native sample had three times higher total phenolic content comparing to the sample with CD. Therefore, the native sample was diluted three times for the assays. Undigested OPE (dissolved in PBS) and OPEs’ bioaccessible fractions were filtered over sterile syringe filter (PES membrane, φ 13 mm, pore size 0.22 μm) while stock solutions of pure polyphenols were diluted with PBS. The stock solutions were added directly to the cell culture medium so the final concentrations of OPE, mix (TS + HTS), TS and HTS in wells are noted in Table 1. Oxidative processes were induced with tBOOH in final concentrations noted in the description of each method [37].

### 3.6. Preparations of Cells for Cell Viability, ROS and GSH Determination

The first part of the cell viability, ROS, and GSH determination assays was similar. Also, all the photometric and fluorometric readings were done on a multilabel plate reader (Victor X3, Perkin Elmer, Waltham, MA, USA). The only difference was the type of the cell culture plates since for the MTT assay the transparent 96-well plates (Thermo Fisher Scientific 130188, Rochester, NY, USA) were used while for the ROS and GSH assays black 96-well plates (Thermo Fisher Scientific 137101, Rochester, NY, USA) were needed. Briefly, 2 × 10^4^ cells/well were seeded in 100 µL of medium in 96-well plates and grown until reaching confluence (approximately 48 h) at 37 °C in a humidity saturated atmosphere consisted of 5% CO_2_. Stock solutions of samples were added directly to the culture medium in volume of 10 µL. Cells were incubated for 20 h with samples or PBS for controls.

Cell viability was determined using the MTT [38] assay to check the toxicity of samples but also to determine the protective effect of samples after induction of oxidative stress with tBOOH. The assay is based on the cleavage of the yellow tetrazolium salt MTT to purple formazan crystals by metabolic active cells. That cellular reduction involves the pyridine nucleotide cofactors NADH and NADPH. The formazan crystals formed are solubilized and the resulting colored solution is quantified spectrophotometrically at 570 nm. After the treatment with samples (as described previously), additional 3-h incubation with 15 µL of 350 µM tBOOH (final concentration in wells) followed only in the assay that determined protective effect (in which positive control wells received 15 µL of PBS instead of tBOOH). The culture medium was then warily aspirated (Gilson Safe Aspiration Station, Middleton, WI, USA), and cells were washed with 250 µL PBS/well. Cell viability was assessed by the addition of 40 µL of MTT 0.5 mg/mL (diluted in PBS) and incubation for 3 h at 37 °C, followed by dissolution of the formazan crystals in 170 µL of DMSO. Absorbance (A) was measured at 490 nm and cell viability was expressed as percentage (%) of the positive control according to Equation (1). Blank absorbance was measured in wells containing MTT without cells:

(1) $$\%\;\mathrm{cell}\;\mathrm{viability}\;=\;\frac{\left(A\;–{\;A}_{b}\right)}{\left(A_{\mathrm{ctr}+}\;–{\;A}_{b}\right)}\times 100$$

Quantitative measurement of the activity of hydroxyl, peroxyl and other reactive oxygen species (ROS) was performed using 2′,7′-dichlorofluorescein diacetate (DCFDA). DCFDA is a fluorescent dye that has the ability to diffuse into a cell where it is deacetylated by cell esterase to form a non-fluorescent compound [39]. Oxidation of this compound with ROS produces highly fluorescent compound 2′,7′-dichlorofluorescein (DCF). DCF can be detected by a fluorescence spectrometer with a maximum excitation/emission spectrum at 495/529 nm. To assess antioxidative activity, the culture medium was warily aspirated (after the 20-h incubation with samples as described previously) and cells were washed with 100 µL of PBS. Then, cells were incubated with 100 µL of 25 µM DCFDA for 40 min. The dye was aspirated, and the cells were washed once again with 100 µL of PBS. Oxidative stress was induced by adding 100 µL of 50 µM tBOOH (diluted in PBS) for 1 h. Positive control wells received 15 µL of PBS instead of tBOOH. Fluorescence (F) was measured at 485/535 nm and % ROS was expressed as relative fluorescence (RFU) of the positive control according to Equation (2). Blank absorbance was measured in wells containing DCFDA without cells.

(2) $$\mathrm{RFU}\;=\;\frac{\left(F\;–{\;F}_{b}\right)}{\left(F_{\mathrm{ctr}+}\;–{\;F}_{b}\right)}\times 100$$

The intracellular concentration of reduced GSH was estimated by fluorometric assay that utilizes monochlorobimane (mBCl). Briefly, GSH reacts with mBCl in an enzyme reaction catalyzed with intracellular glutathione S-transferases. Adducts that form in that reaction can be measured fluorometrically at an excitation wavelength of 380 nm and an emission wavelength of 470 nm using a microplate reader [40]. After the 20-h incubation with samples (as described previously), 3-h incubation with prooxidant was done by adding 15 µL of 50 µM tBOOH (final concentration in wells). Positive control wells received 15 µL of PBS instead of tBOOH. The culture medium was then warily aspirated, and cells were washed with 100 µL of PBS after which 40-min incubation with 100 µL of 40 µM mBCl followed. Fluorescence (F) was measured at 355/460 nm and % GSH was expressed as relative fluorescence (RFU) of the positive control according to Equation (2). Blank absorbance was measured in wells containing mBCl without cells.

To assess the effect of olive pomace polyphenols on GPx and SOD activity, 10^6^ cells were seeded in each well in 5 mL of culture medium in 6-well Falcon plate (Corning Inc., Corning, NY, USA). Cells were grown until reaching confluence (approximately 48 h) at 37 °C in a humidity saturated atmosphere consisted of 5% CO_2_. Samples were added directly to the culture medium in volume of 500 µL. Cells were incubated for 20 h with samples or PBS for control. Then the cells were washed with 1 mL of PBS and lysed with 200 µL of the lysis buffer in 20 min placed on ice. Cells were scraped, placed in Eppendorf tubes and centrifugated at 15000 G for 20 min at 4 °C. Supernatant were collected and used for protein quantification and enzyme activity determination.

Protein concentration was determined by BCA method which is based on complex formation between protein and Cu^2+^ in alkali conditions. In those conditions, Cu^2+^ is reduced to Cu^+^ that forms a purple complex with BCA that can be detected by measuring absorbance at 570 nm. The assay was done in 96-well plates by adding 4 µL of cell lysate, 21 µL of water and 100 µL of working reagent. The plates were shaken slowly for 1 min in a multiplate reader and incubated at 37 °C, 30 min in total, after which the absorbance was measured at 490 nm. Blank contained 25 µL of water instead of samples and the obtained value was deducted from all the samples’ values. The protein concentration was determined by comparing the result with bovine serum albumin (BSA) using the calibration curve made in the same conditions. The concentration range of BSA was 0.1–1 mg/mL prepared in aqua pro injectione.

GPx activity was measured indirectly by a coupled reaction with glutathione reductase. Oxidized glutathione (GSSG), produced upon reduction of hydroperoxide by GPx, is recycled to its reduced state by GR and NADPH. The oxidation of NADPH to NADP^+^ is accompanied by a decrease in absorbance at 340 nm. Under conditions in which the GPx activity is rate limiting, the rate of decrease in A_340_ is directly proportional to the GPx activity in the sample [41]. One unit was defined as the amount of enzyme that will cause the oxidation of 1 nmol of NADPH to NADP^+^ per minute at 25 °C. Briefly, 20 µL of cell lysate was mixed with 50 µL of Assay Buffer, 50 µL of Co-Substrate Mixture, and 50 µL of NADPH after which the reaction was initiated by adding 20 µL of Cumene Hydroperoxide. The plate was slowly mixed for 5 s in a multiplate reader at room temperature and the absorbance was measured every minute for 10 min. The absorbance decreased linearly over time, so the specific enzyme activity was calculated as noted in Equation (3):

(3) $$\mathrm{GPx}\;\mathrm{activity}\;\left(\mathrm{nmol}/\min/\mathrm{mg}\right)=\frac{|\mathrm{slope}\;(\min^{-1})|\times {\;V}_{t}\;\left(\mathrm{mL}\right)}{\varepsilon \;({\text{µ}M}^{-1})\times \;V\;\left(\mathrm{mL}\right)}$$

where slope is from the A_340_ decrease over time, V_t_ is the total volume in the well, V is the volume of the sample, ε is the NADPH extinction coefficient at 340 nm of 0.00373 µM^−1^ (the value is adjusted for the pathlength of 0.6 cm of solution in the well).

SOD activity was measured by indirect method using Dojindo’s tetrazolium salt WST-1 (2-(4-iodophenyl)-3-(4-nitrophenyl)-5-(2,4-disulfophenyl)-2H-tetrazolium, monosodium salt). WST-1 produces a water-soluble formazan dye upon reduction with a superoxide anion that absorbs light at 440 nm. Since the absorbance is proportional to the amount of superoxide anion, the SOD activity as an inhibition rate can be quantified by measuring the decrease in the color development at 440 nm [42]. Briefly, 20 µL of cell lysate was mixed with 200 µL of WST Working Solution and 20 µL of Enzyme Working Solution in 96-well plate. The plate was incubated at 37 °C in a multilabel plate reader for 20 min and the absorbance was read at 450 nm. The control wells received water instead of sample. The SOD activity expressed as inhibition rate was calculated according to the Equation (4).

(4) $$\mathrm{SOD}\;\mathrm{activity}\;\left(\mathrm{inhibition}\;\mathrm{rate}\;\left(\%\right)\right)=\frac{\left(A_{b20}-A_{b0})-(A_{20}-A_{0}\right)}{\left(A_{b20}-A_{b0}\right)}\times 100$$

where b represents the blank well in which water was added instead of cell lysate, 0 and 20 are times of measurement.

## Figures and Tables

**Table 1:** Final concentrations of the samples in wells.

	γ_OPE_ (mg/mL)	γ_HTS_ (µM)	γ_TS_ (µM)
nat	0.8	-	-
nat_bf	0.8	-	-
hpbCD	2.5	-	-
hpbCD_bf	2.5	-	-
mix	-	3.7	1.2
TS	-	-	20
HTS	-	20	-

OPE (olive pomace extract), nat (native OP), hpbCD (OP + hydroxypropyl β CD), bf (bioaccessible fraction), TS (tyrosol), HTS (hydroxytyrosol), mix (HTS + TS).

## Supplementary Materials

The following are available online, Figure S1: Effect of a 20-hour exposure of olive pomace extracts and pure polyphenols on HepG2 cell viability, Figure S2: Bioaccessibility of hydroxytyrosol (HTS) and tyrosol (TS) from olive pomace extracts, Figure S3: Chromatograph of olive pomace extract, Figure S4: Total phenolic content determined by Folin-Ciocalteu method.
