# Supplementary material for: Cellular Antioxidant Activity of Olive Pomace Extracts: Impact of Gastrointestinal Digestion and Cyclodextrin Encapsulation
Source: Molecules. 2020 Oct 29;25(21):5027. doi: 10.3390/molecules25215027 (PMC7663658; doi:10.3390/molecules25215027)
Supplement: Supplementary file 1 [file molecules-25-05027-s001.zip › Figure S2.pdf]

Examined parameters	nat	hpbCD
**HTS (μg/100 mg)**		
**undigested extract**	71.7	36.0
**bioaccessible fraction**	67.8	34.4
**TS (μg/100 mg)**		
**undigested extract**	23.5	8.9
**bioaccessible fraction**	30.4	15.0
