# Supplementary figures and images for: Cellular Antioxidant Activity of Olive Pomace Extracts: Impact of Gastrointestinal Digestion and Cyclodextrin Encapsulation
Source: Molecules. 2020 Oct 29;25(21):5027. doi: 10.3390/molecules25215027 (PMC7663658; doi:10.3390/molecules25215027)

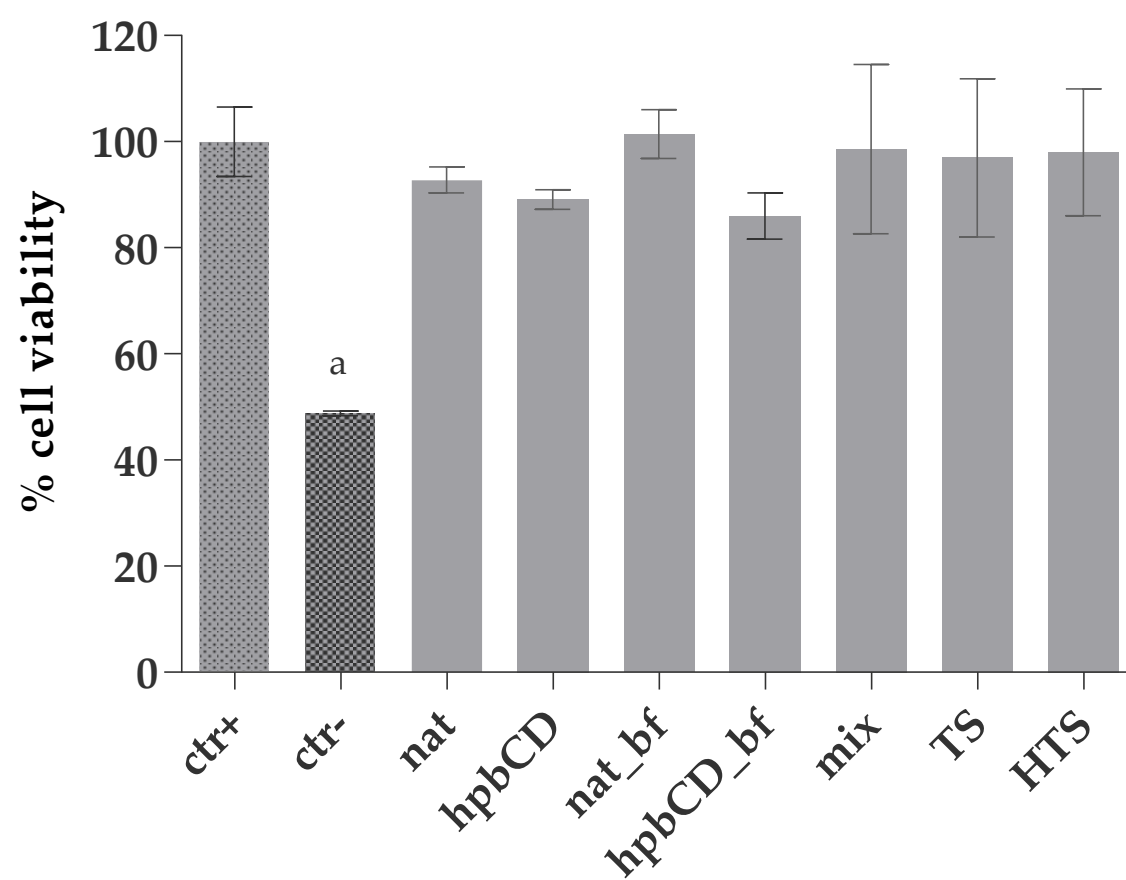

Supplement: Supplementary file 1 [file molecules-25-05027-s001.zip › Figure S1.pdf]

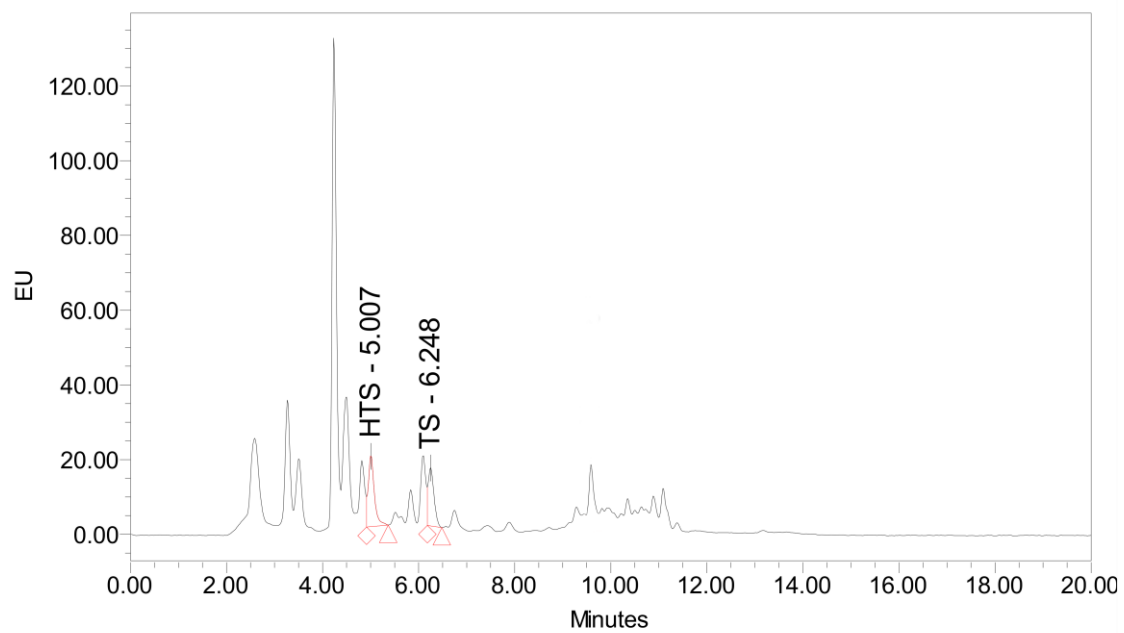

Supplement: Supplementary file 1 [file molecules-25-05027-s001.zip › Figure S3.pdf]

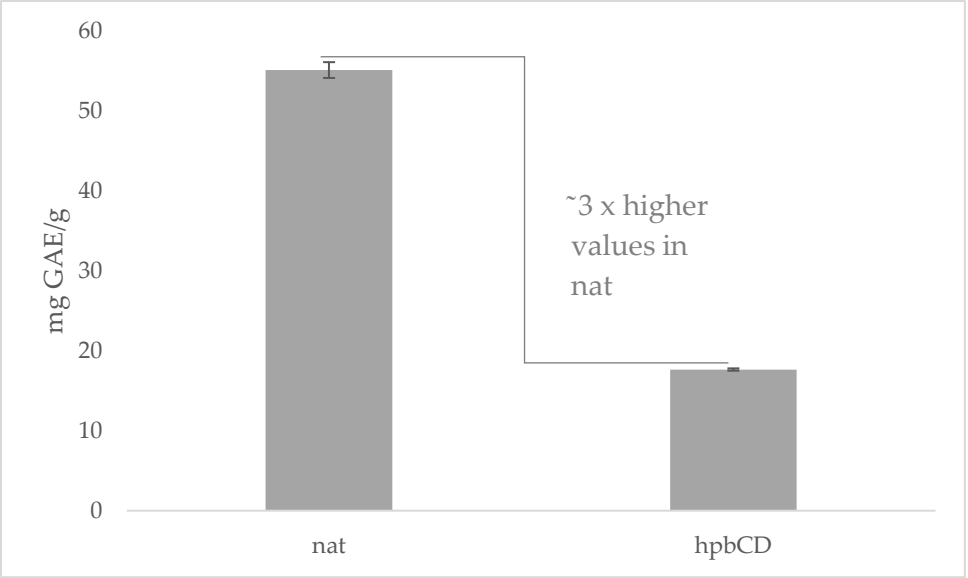

Supplement: Supplementary file 1 [file molecules-25-05027-s001.zip › Figure S4.pdf]
